# Physiological and biochemical changes during drought and recovery periods at tillering and jointing stages in wheat (*Triticum aestivum* L.)

**DOI:** 10.1038/s41598-018-21441-7

**Published:** 2018-03-15

**Authors:** Muhammad Abid, Shafaqat Ali, Lei Kang Qi, Rizwan Zahoor, Zhongwei Tian, Dong Jiang, John L. Snider, Tingbo Dai

**Affiliations:** 1 Key Laboratory of Crop Physiology, Ecology and Production Management, Nanjing Agricultural University, Nanjing 210095, Jiangsu Province, 210095 P. R. China; 2 Department of Soil and water Conservation, Directorate General of Field, Narowal, 51800 Punjab Pakistan; 3 Department of Environmental Sciences and Engineering, Allama Iqbal Road 38000, Government College University, Faisalabad, Pakistan; 4 Department of Crop and Soil Sciences, University of Georgia, Tifton, Georgia 31794 USA

## Abstract

Defining the metabolic strategies used by wheat to tolerate and recover from drought events will be important for ensuring yield stability in the future, but studies addressing this critical research topic are limited. To this end, the current study quantified the physiological, biochemical, and agronomic responses of a drought tolerant and drought sensitive cultivar to periods of water deficit and recovery. Drought stress caused a reversible decline in leaf water relations, membrane stability, and photosynthetic activity, leading to increased reactive oxygen species (ROS) generation, lipid peroxidation and membrane injury. Plants exhibited osmotic adjustment through the accumulation of soluble sugars, proline, and free amino acids and increased enzymatic and non-enzymatic antioxidant activities. After re-watering, leaf water potential, membrane stability, photosynthetic processes, ROS generation, anti-oxidative activities, lipid peroxidation, and osmotic potential completely recovered for moderately stressed plants and did not fully recover in severely stressed plants. Higher photosynthetic rates during drought and rapid recovery after re-watering produced less-pronounced yield declines in the tolerant cultivar than the sensitive cultivar. These results suggested that the plant’s ability to maintain functions during drought and to rapidly recover after re-watering during vegetative periods are important for determining final productivity in wheat.

## Introduction

Within an agricultural context, drought is a prolonged period of deficient precipitation which results in negative impacts on crop growth or yield. An increasingly warming climate is expected to intensify the frequency and severity of drought in the near future^1^. Thus, identifying key physiological limitations to productivity under drought and mechanisms of crop tolerance to water deficit stress will be important for improving yield stability in a changing climate. Moreover, limited genetic diversity within important crop species coupled with ecological constraints to productivity need to be overcome in-order to adapt crops to episodic drought events in the future^2,3^. The ability of plants to maintain physiological functions at low plant water status and recover quickly once the stress is removed will be important for ensuring sustainable crop production under intermittent drought events^4^. The effects of drought stress have been well-documented in many crop species; however, reports addressing physiological responses to progressive drought and recovery upon re-watering are relatively limited^3^.

Reduced plant growth and productivity under drought are caused by altered plant water relations, decreased CO_2_ assimilation, cellular oxidative stress, membrane damage of affected tissues, and in some instances, inhibition of enzyme activity. Plants respond to drought stress by exploiting the following mechanisms: (1) drought escape by completing the life cycle before the onset of severe water limitation^4^; (2) drought avoidance through an enhanced water conserving mechanism via stomatal closure and reduction of leaf area or canopy cover^1^; (3) drought tolerance through osmotic adjustment and increased cell wall elasticity^5^, and (4) drought resistance through altered metabolic changes such as an increased antioxidant metabolism^6^. Plants can employ the above-mentioned mechanisms in response to drought stress consecutively or simultaneously.

Under drought stress, reductions in carbon assimilation result in an imbalance between electron excitation and utilization through photosynthesis, which results in the production of reactive oxygen species (ROS), primarily superoxide (O_2_^•−^) and hydrogen peroxide (H_2_O_2_)^6^. These ROS damage cell membranes, proteins, and nucleic acids, causing oxidative stress^7^. The intercellular concentration of malondialdehyde (MDA) indicates the extent of oxidative stress^8^. The plants possess enzymatic and non-enzymatic mechanisms to detoxify ROS^9^. Superoxide dismutase (SOD) catalyzes the conversion of O_2_^•−^ to the less reactive H_2_O_2_^10^. This H_2_O_2_ is further detoxified to O_2_ and H_2_O through the activities of catalase (CAT) and ascorbate peroxidase (APX). Combined, the three enzymes noted above ensure low intracellular levels of O_2_^−^ and H_2_O_2_^11^. Glutathione (GSH) and carotenoids are among the non-enzymatic antioxidants involved in cellular defense^12^. GSH protect the chloroplasts from ROS damage by increasing the ratio of reduced glutathione to oxidized glutathione (GSH/GSSG)^13^, whereas carotenoids safeguard the photosynthetic apparatus by dissipating excess excitation energy into heat^14^.

Similarly, under drought conditions, plants can alter water relations to maintain cellular functions^4^. For example, plants exhibit osmotic adjustment by synthesizing and accumulating compatible solutes such as free amino acids, sugars, and proline^15^. Osmotic adjustment allows the plant to maintain turgor pressure and cell volume at low water potential, which is important for maintaining metabolic functions^4,16^. In addition, osmotic adjustment facilitates the recovery of metabolic activities after relief from stress^16^.

Although attempts have been made to investigate the recovery of photosynthesis from drought stress in different crop species including wheat^3,5,16^, studies addressing membrane stability, oxidative stress, antioxidative process, and osmolyte dynamics during drought recovery are limited. Moreover, studies quantifying the impact of plant metabolic changes during drought and recovery periods during vegetative development on final productivity in wheat are, to our knowledge, non-existent because physiological changes during reproductive stages are understandably related to grain yields and have received far more attention^17^. Nonetheless, stress events during vegetative growth periods can significantly influence grain yield of wheat and should be investigated further^17^.

After drought stress is removed, the availability of even a small amount of rainfall can have a significant effect on plant physiological functions, ranging from whole-plant responses to biochemical responses. Therefore, it is of particular importance to investigate the underlying mechanisms contributing to drought tolerance^4^. We hypothesized that 1) final productivity in wheat would be dependent on the ability to maintain photosynthetic stability under drought stress and to rapidly recover to pre-drought levels upon rewatering, 2) the ability to osmotically adjust and protect cellular components from oxidative stress will be critical factors influencing tolerance to episodic drought during the vegetative phase. The present experiments were carried out to quantify physiological and yield responses of wheat cultivars to episodic drought and re-watering when they were subjected to different intensities of drought during vegetative growth stages.

## Results

**Figure 1:**
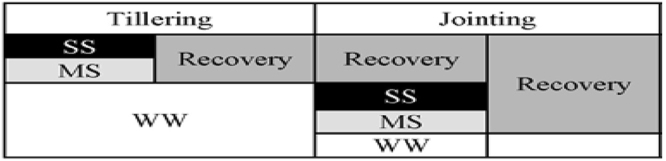
Experimental design of the study: drought stress treatments were applied during tillering and jointing stages by withholding irrigation till the soil field capacity (FC) reached 35–40% and 55–60% for severe stress (SS) and moderate stress (MS), respectively. The drought treatments were maintained for 10 days by weighing the pots and compensating the water lost to the desired FC and then followed by re-watering at 75–80% FC. Severe and moderate water deficit treatments during tillering and jointing were designated as SS and MS, respectively, while, well-watered (control) was designated as WW treatments.

Two wheat cultivars, Luhan7 and Yangmai16, hereafter referred to as ‘tolerant’ and ‘sensitive’, were exposed to severe and moderate soil water stresses during tillering and jointing stages, respectively, followed by re-watering (Fig. 1) to quantify the metabolic changes associated with drought tolerance in wheat.

### Amount of water applied

An average amount of water applied to plants of Luhan-7 and Yangmai-16 during the drought periods in both growing seasons under different drought treatments is given in Table 1. During severe (SS) and moderate stress (MS) applications, Yangmai-16 used less amount of water than Luhan-7 as compared to WW treatment.

**Table 1:** Amount of water (L pot^−1^) used by plants during the stress periods applied at tillering and jointing stages of Luhan-7 and Yangmai-16 wheat cultivars.

Growth stages	Tillering						Jointing					
Water deficits	SS		MS		WW		SS		MS		WW	
Cultivars	LH-7	YM-16	LH-7	YM-16	LH-7	YM-16	LH-7	YM-16	LH-7	YM-16	LH-7	YM-16
2014–2015	0.7	0.5	1	0.9	1.2	1.5	1.1	0.9	1.3	1.1	1.4	1.7
2015–2016	0.8	0.6	1.1	0.9	1.3	1.5	1.3	0.9	1.4	1	1.6	1.6

Drought stress was applied during tillering and jointing, respectively followed by re-watering. SS: severe stress, MS: moderate stress, WW: well-watered, and LH-7 and YM-16 were abbreviated for Luhan-7 and Yangmai-16 wheat cultivars, respectively.

### Changes in net photosynthetic rate and stomatal conductance

**Figure 2:**
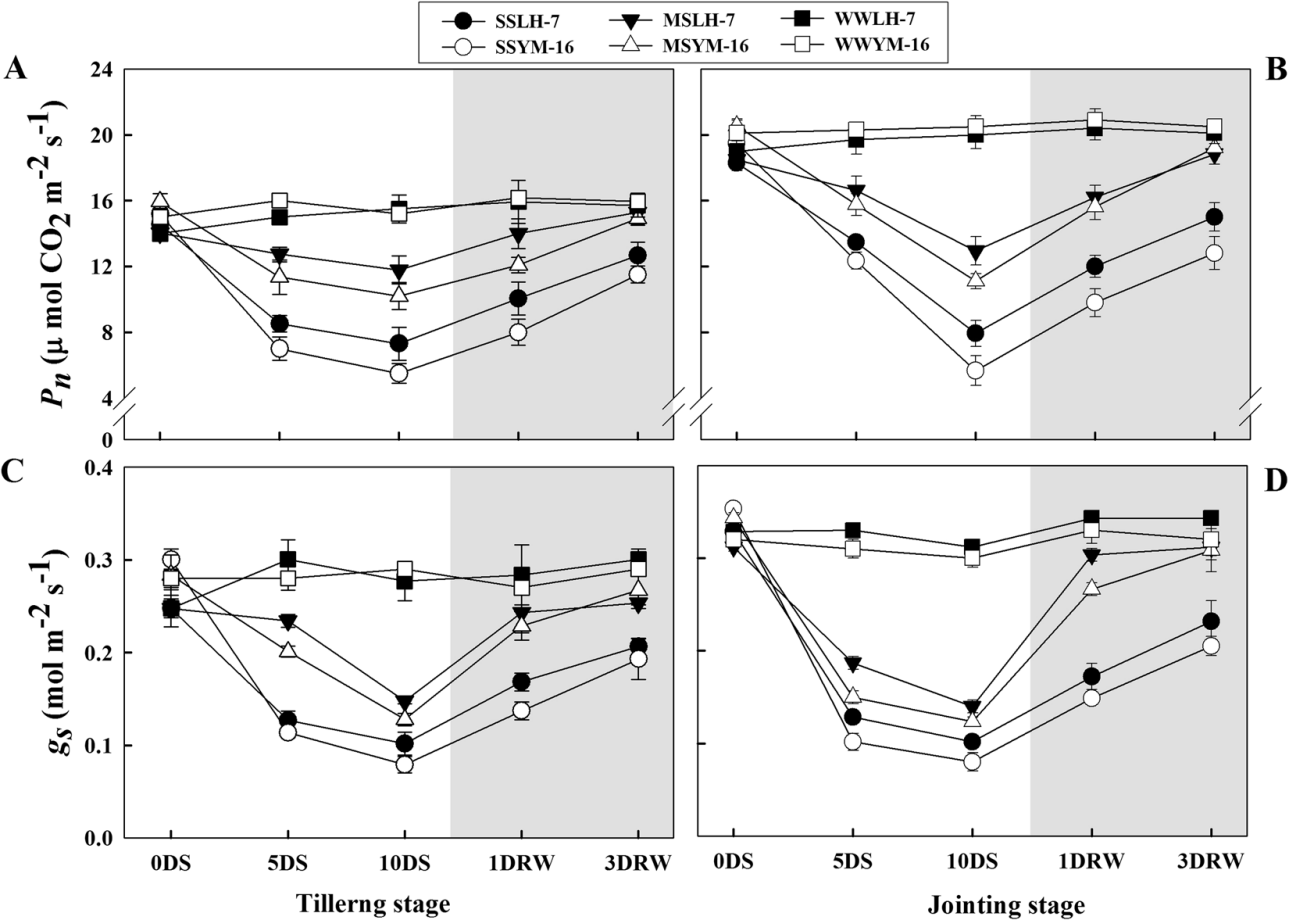
Effect of drought stress (SS: severe stress, MS: moderate stress and WW: well watered) on net photosynthetic rate (Pn) (**A,B**) and stomatal conductance (gs) (**C,D**) in Luhan7 (LH-7) and Yangmai16 (YM-16) wheat cultivars. SS and MS treatments were applied at 35–40% and 55–60% soil field capacity (FC), respectively for ten days at tillering and jointing growth stages followed by re-watering, whereas WW was maintained at 75–80% FC. Time-course of the measurements was one day before stress (0DS), 5^th^ and 10^th^ day of stress (5DS, 10DS), 1 and 3 days after re-watering (1DRW, 3DRW). Shaded areas indicate the measurements taken following re-watering. Each vertical bar above the means indicates standard error of six replicates (*n* = 6) by using two-way ANOVA at P < 0.05.

Figure 2 shows that drought stress induced a gradual decline in net photosynthetic rate (Pn) and stomatal conductance (gs) as compared to WW plants during the stress period. The magnitude of decline was greater (P ≤ 0.05) for plants under severe drought stress (SS) during the jointing stage than moderate stress (MS) during tillering in both cultivars. During both stages, under SS and MS treatments, the Pn and gs decreased more so in the sensitive cultivar than the drought tolerant cultivar when compared to WW plants. After re-watering, the plants showed a progressive increase in Pn and gs and recovered fully to the levels of the WW treatment under MS treatments at three days after re-watering (3DRW). By comparison only partial recovery of Pn and gs was noted for the SS treatments following the same recovery period. Plants from both stress treatments showed greater recovery when stress was imposed in the tillering stage than the jointing stage in the two cultivars.

### Changes in membrane stability index and membrane injury

**Figure 3:**
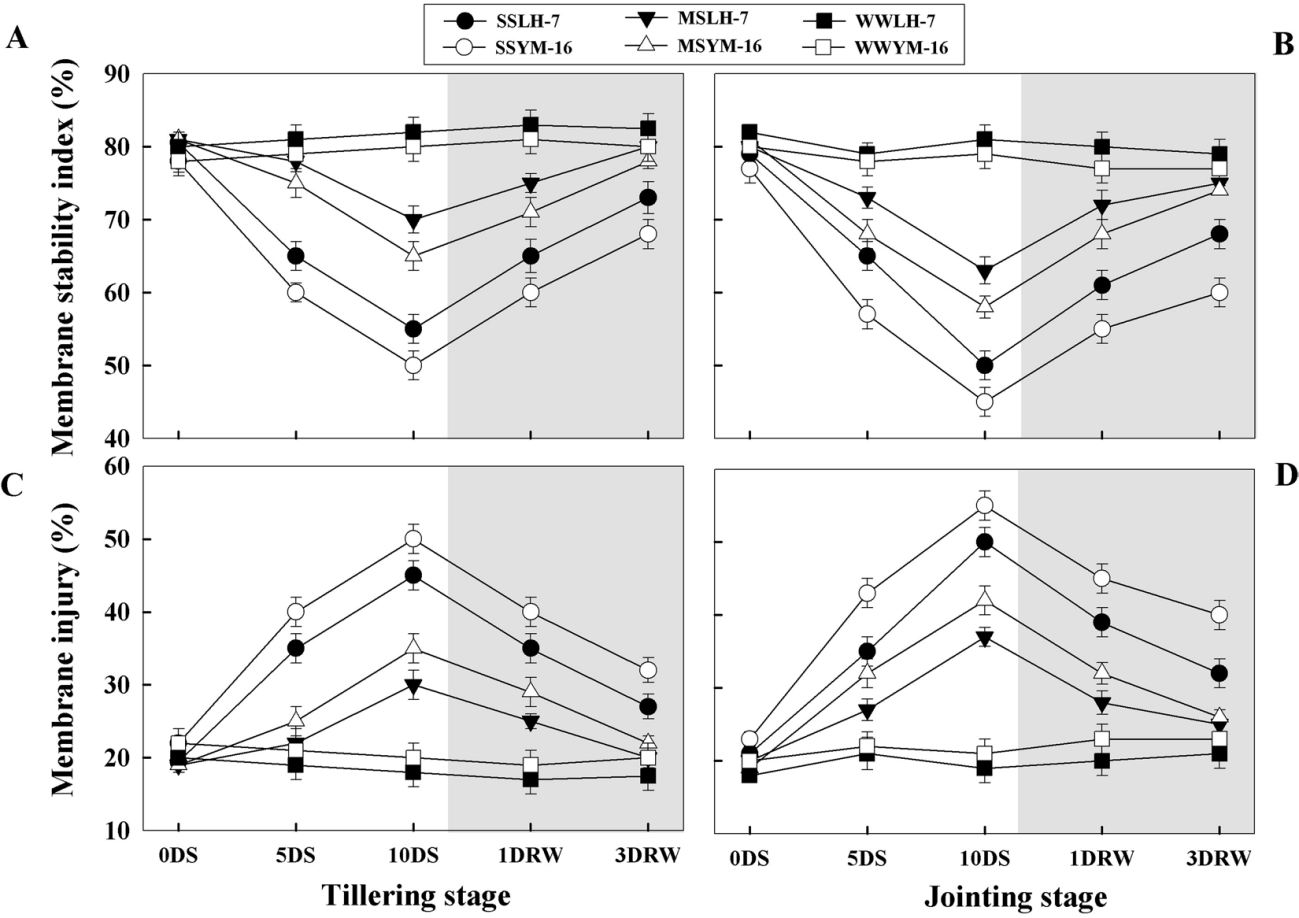
Effect of drought stress (SS: severe stress, MS: moderate stress and WW: well watered) on membrane stability index (MSI) (**A,B**) and membrane injury (MI) (**C,D**) in Luhan7 (LH-7) and Yangmai16 (YM-16) wheat cultivars. SS and MS treatments were applied at tillering and jointing growth stage at 35–40% and 55–60% soil field capacity (FC), respectively for ten days followed by re-watering, whereas WW was maintained at 75–80% FC. Time-course of the measurements was one day before stress (0DS), 5^th^ and 10^th^ day of stress (5DS, 10DS), 1 and 3 days after re-watering (1DRW, 3DRW). Shaded areas indicate the measurements taken following re-watering. Each vertical bar above the means indicates standard error of six replicates (*n* = 6) by using two-way ANOVA at P < 0.05.

Drought stress caused a decrease in membrane stability index (MSI) (Fig. 3A,B) and an increase in membrane injury (MI) (Fig. 3C,D) during the stress period. The magnitude of decline in MSI was greater (P ≤ 0.05) for plants under SS than that under MS treatments and it was more pronounced at jointing than at the tillering stage. During stress periods, tolerant plants maintained significantly (P ≤ 0.05) higher MSI and exhibited lower MI as compared to the sensitive cultivar. After re-watering, both MSI and MI recovered progressively to WW levels by 3DRW in MS plants, whereas the SS plants showed incomplete recovery within the same rewatering time frame. MSI and MI showed similar recovery trends, irrespective of the growth stage at which stress was imposed.

### Changes in reactive oxygen species and MDA contents

**Figure 4:**
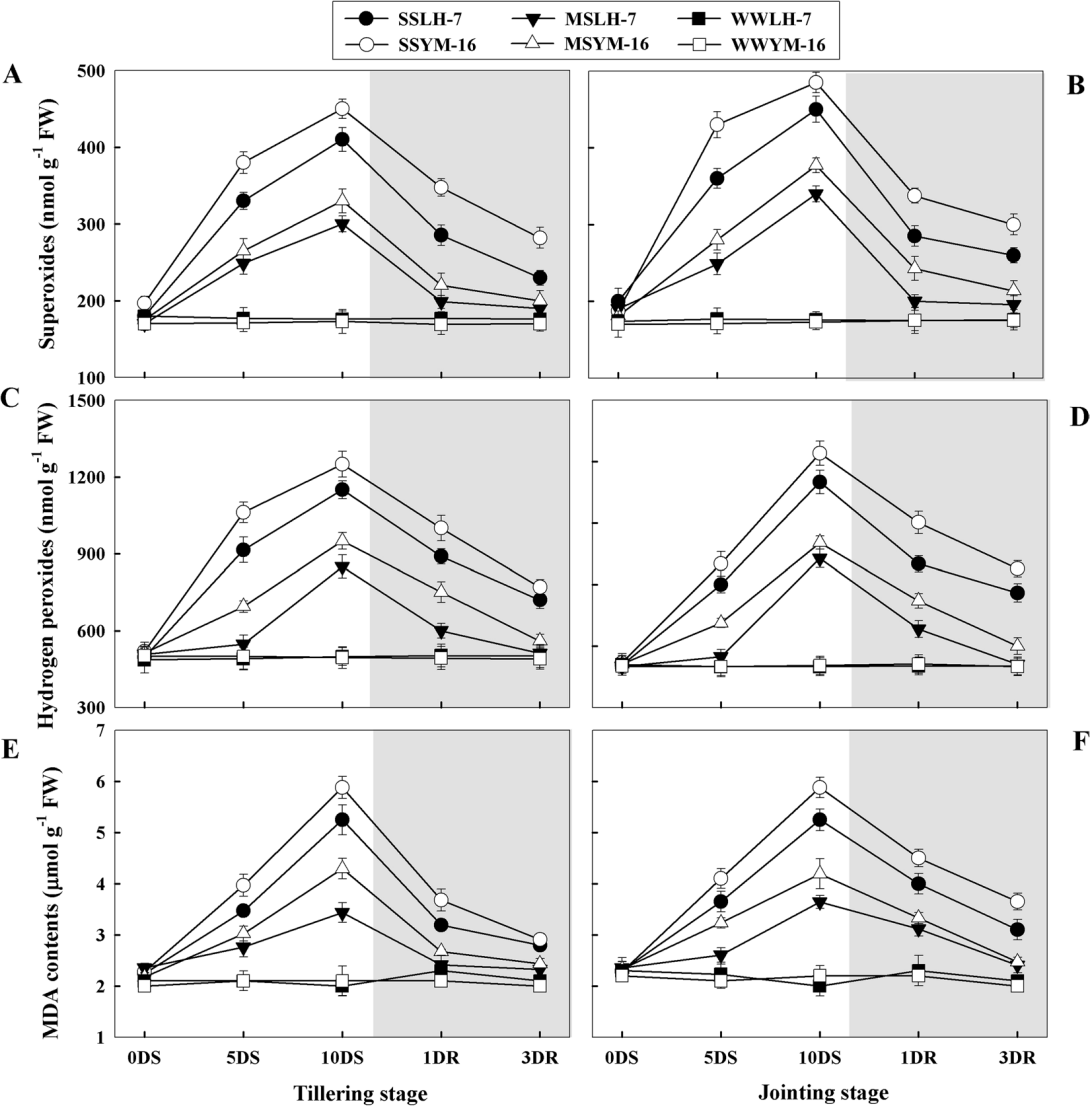
Effect of drought stress (SS: severe stress, MS: moderate stress and WW: well watered) on the production of superoxides (**A,B**), hydrogen peroxide (**C,D**) and MDA contents (**E,F**) in Luhan7 (LH-7) and Yangmai16 (YM-16) wheat cultivars. SS and MS treatments were applied at tillering and jointing growth stage at 35–40% and 55–60% soil field capacity (FC), respectively for ten days followed by re-watering, whereas WW was maintained at 75–80% FC. Time-course of the measurements was one day before stress (0DS), 5^th^ and 10^th^ day of stress (5DS, 10DS), 1 and 3 days after re-watering (1DRW, 3DRW). Shaded areas indicate the measurements taken following re-watering. Each vertical bar above the means indicates standard error of six replicates (*n* = 6) by using two-way ANOVA at P < 0.05.

The contents of O_2_^•−^, H_2_O_2_ and MDA increased rapidly in SS and more moderately in MS as compared with WW plants (Fig. 4). The same trend for the generation of O_2_^•−^, H_2_O_2_ and MDA was observed during both growth stages; however, the magnitude of increase in the concentrations of these substances due to drought stress was lower during tillering than at jointing. Drought-stressed plants of the sensitive cultivar contained higher contents of O_2_^•−^, H_2_O_2_ and MDA than did plants of the tolerant cultivar, irrespective of growth stage. After re-watering, the concentration of O_2_^•−^, H_2_O_2_ and MDA decreased rapidly, especially in MS plants, which reached levels comparable to WW plants on 1DRW for the tolerant cultivar and on 3DRW in both cultivars. For SS plants, contents of O_2_^•−^, H_2_O_2_ and MDA never fully declined to the level of WW plants, even after 3 DRW.

### Changes in enzymatic antioxidant activities

**Figure 5:**
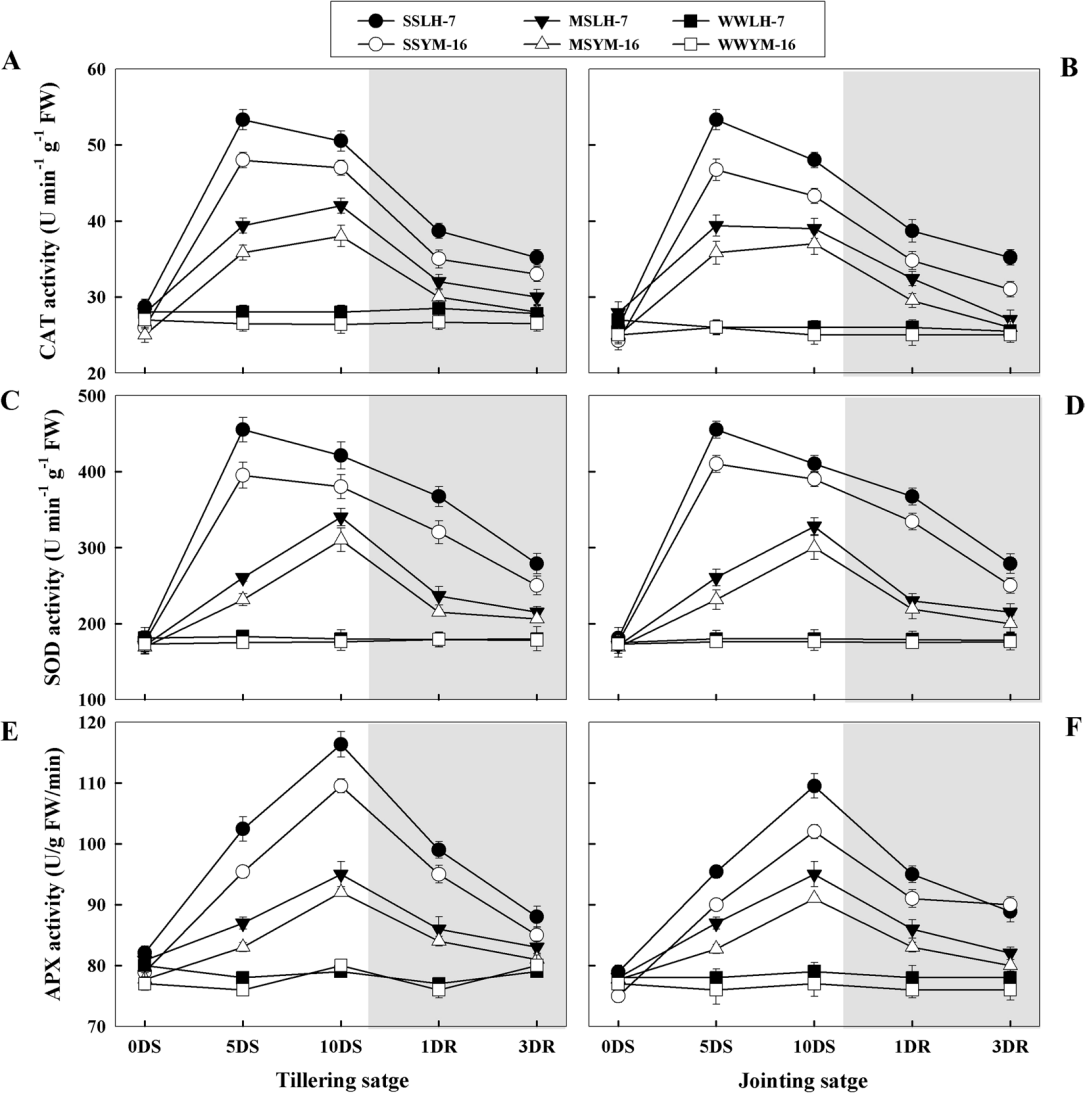
Effect of drought stress (SS: severe stress, MS: moderate stress and WW: well watered) on CAT (catalyses) (**A**,**B**), SOD (superoxidase dismutase) (**C,D**), and APX (ascorbate peroxidases) (**E,F**) activities in Luhan7 (LH-7) and Yangmai16 (YM-16) wheat cultivars. SS and MS treatments were applied at 35–40% and 55–60% soil field capacity (FC), respectively for ten days at tillering and jointing growth stage followed by re-watering, whereas WW was maintained at 75–80% FC. Time-course of the measurements was one day before stress (0DS), 5^th^ and 10^th^ day of stress (5DS, 10DS), 1 and 3 days after re-watering (1DRW, 3DRW). Shaded areas indicate the measurements taken following re-watering. Each vertical bar above the means indicates standard error of six replicates (*n* = 6) by using two-way ANOVA at P < 0.05.

During drought stress, the enzymatic activities of CAT, SOD, and APX increased (Fig. 5). A rapid increase in the activities of CAT and SOD was observed, which reached a maximum at 5DS, whereas, APX activity reached a maximum on last day of drought stress (10DS). Overall antioxidant enzyme activities were higher under SS than MS without any significant difference (P ≤ 0.05) between growth stages except for APX, which exhibited higher activity during the tillering stage than the jointing stage under SS. The tolerant cultivar exhibited higher antioxidant enzyme activity than the sensitive cultivar at both growth stages (P ≤ 0.05). After re-watering, CAT, SOD and APX activities decreased but remained higher in SS plants as compared to WW plants, even after 3DRW. By comparison, in MS plants, enzyme activities recovered to the level of WW plants on 3DRW for both cultivars.

### Changes in non-enzymatic antioxidants

**Figure 6:**
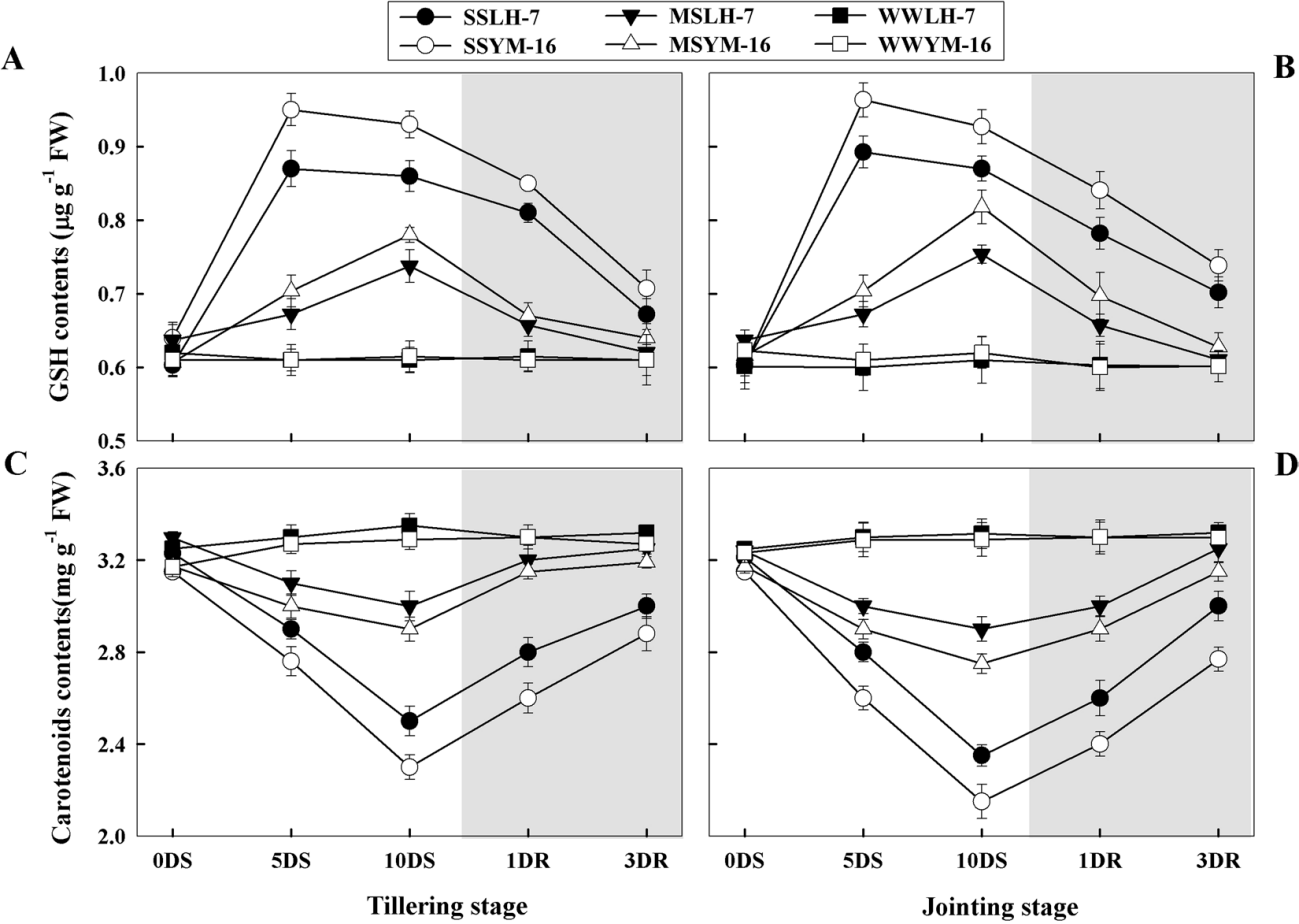
Effect of drought stress (SS: severe stress, MS: moderate stress and WW: well watered) on GSH (reduced glutathione) (**A,B**) and carotenoids (**C,D**) contents in Luhan7 (LH-7) and Yangmai16 (YM-16) wheat cultivars. SS and MS treatments were applied at 35–40% and 55–60% soil field capacity (FC), respectively for ten days at tillering and jointing growth stages followed by re-watering, whereas WW was maintained at 75–80% FC. Time-course of the measurements was one day before stress (0DS), 5^th^ and 10^th^ day of stress (5DS, 10DS), 1 and 3 days after re-watering (1DRW, 3DRW). Shaded areas indicate the measurements taken following re-watering. Each vertical bar above means indicates standard error for six replicates. Each vertical bar above the means indicates standard error of six replicates (*n* = 6) by using two-way ANOVA at P < 0.05.

At both levels of drought stress, a rapid increase in the content of GSH was observed during the early days of stress. As drought progressed, a decline in the pool of GSH occurred in both cultivars at both stages (Fig. 6A,B). The sensitive cultivar showed significantly (P ≤ 0.05) higher GSH contents than the tolerant cultivar both at tillering and jointing under SS and MS treatments. After re-watering, the GSH contents decreased to the level of WW plants under MS but remained higher under SS than under WW conditions. Carotenoid content of the stressed plants showed a decreasing trend with a greater reduction in content under SS than MS (Fig. 6C,D). Under drought stress, the magnitude of reduction in carotenoids was significantly greater (P ≤ 0.05) for the sensitive cultivar than for the tolerant cultivar and this effect was most pronounced at the jointing stage. After re-watering, the carotenoid content tended to increase and reached levels comparable to WW plants by 3DRW for plants under MS, whereas carotenoid content remained lower than WW plants for the SS treatment, even after 3DRW.

### Changes in contents of proline, amino acids and soluble proteins

**Figure 7:**
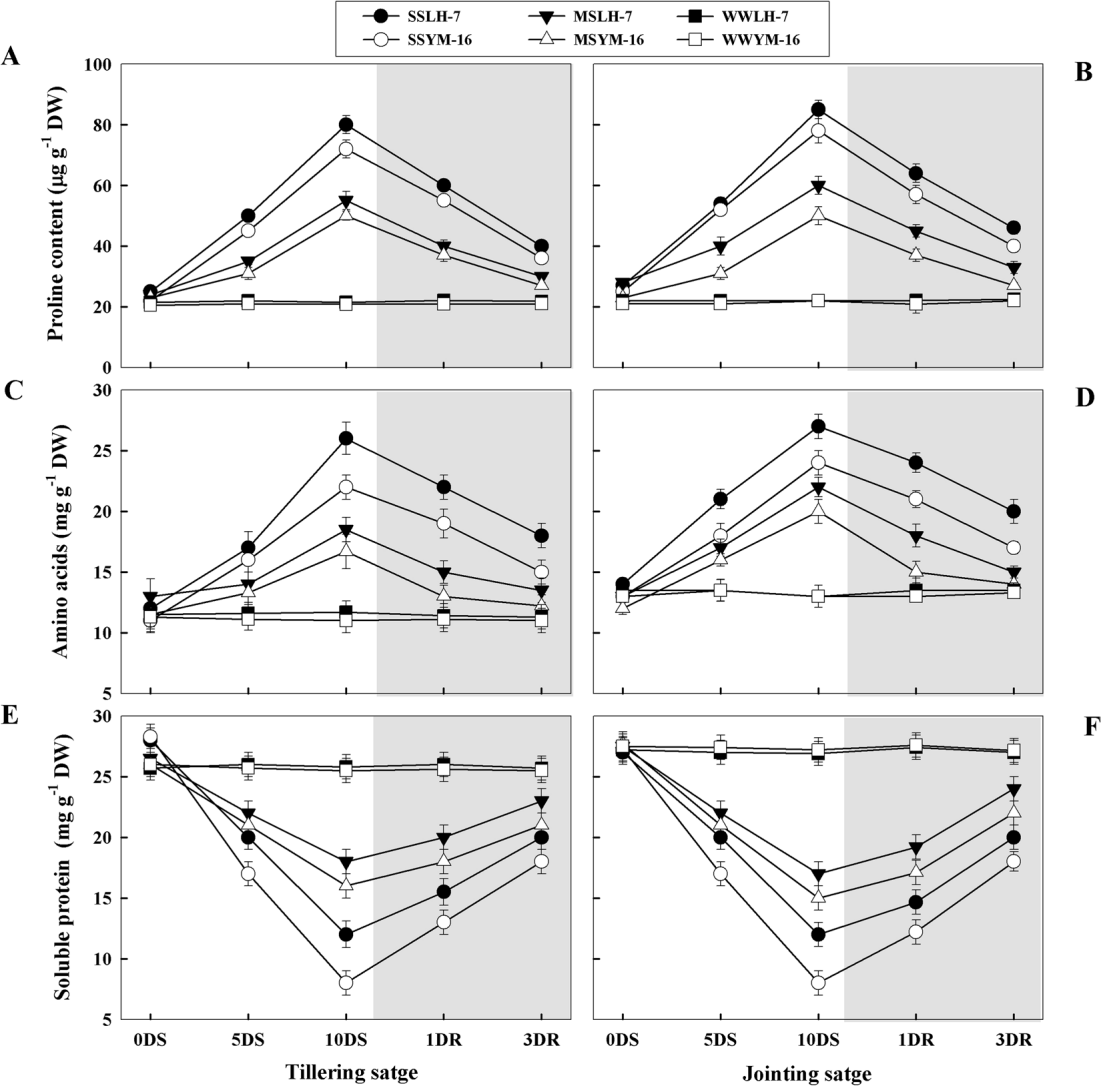
Effect of drought stress (SS: severe stress, MS: moderate stress and WW: well watered) on soluble protein (**A,B**), amino acid (**C,D**) and proline (**E,F**) production in Luhan7 (LH-7) and Yangmai16 (YM-16) wheat cultivars. SS and MS treatments were applied at 35–40% and 55–60% soil field capacity (FC), respectively for ten days at tillering and jointing growth stages followed by re-watering, whereas WW was maintained at 75–80% FC. Time-course of the measurements was one day before stress (0DS), 5^th^ and 10^th^ day of stress (5DS, 10DS), 1 and 3 days after re-watering (1DRW, 3DRW). Shaded areas indicate the measurements taken following re-watering. Each vertical bar above the means indicates standard error of six replicates (*n* = 6) by using two-way ANOVA at P < 0.05.

Under drought stress, the concentration of soluble protein decreased and that of free amino acids and proline increased (Fig. 7). A greater increase in amino acids and proline and conversely, a greater decrease in soluble protein content were observed under SS than MS treatments. The sensitive cultivar showed a lower magnitude of increase in amino acid and proline concentration and a higher reduction in soluble protein than the tolerant cultivar. After re-watering, the concentrations of all free amino acids, proline, and protein tended to return to well-watered levels, but more rapid recovery was observed under MS than SS treatments in both cultivars.

### Changes in carbohydrates concentrations

**Figure 8:**
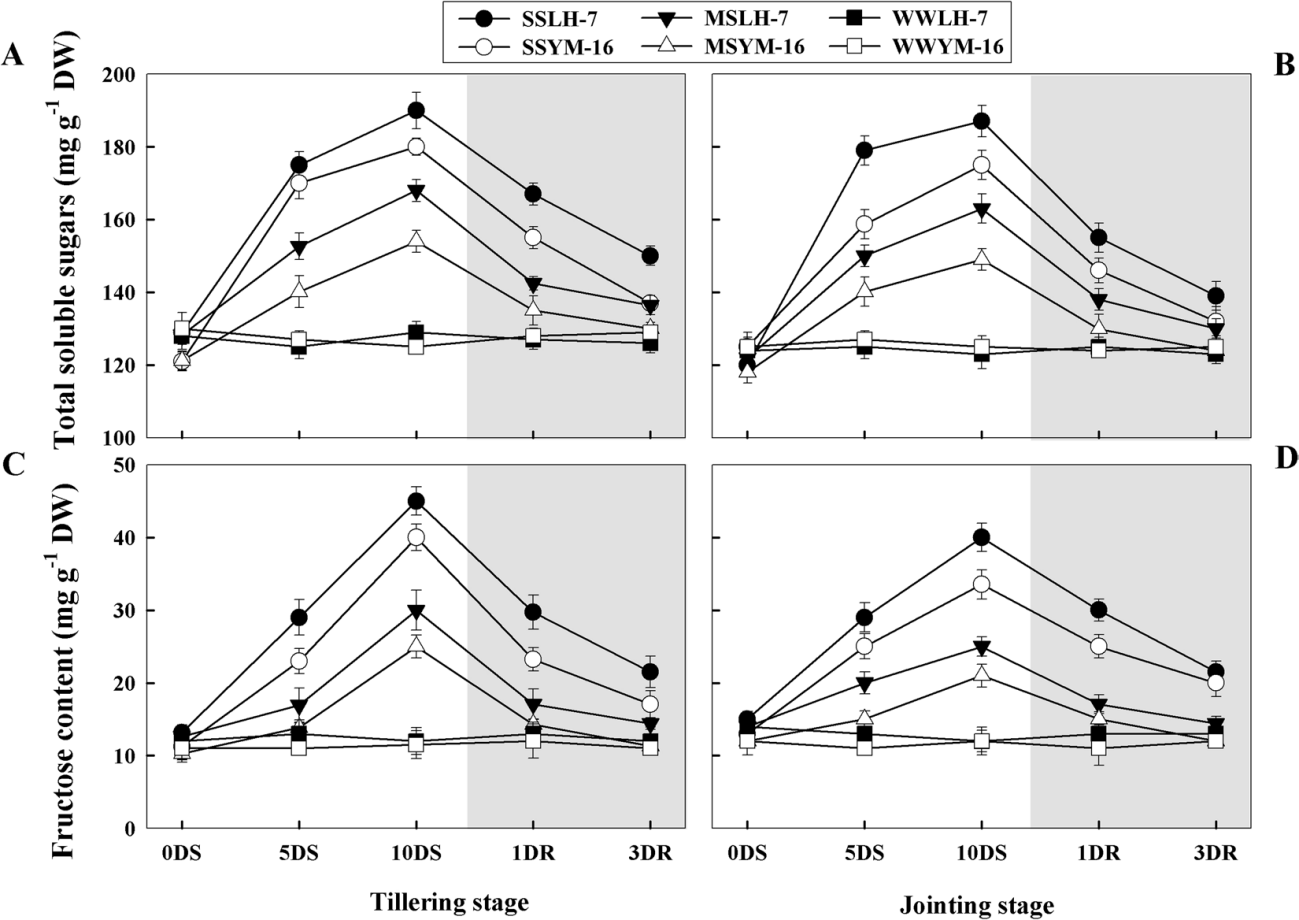
Effect of drought stress (SS: severe stress, MS: moderate stress and WW: well watered) on total soluble sugars (**A,B**) and fructose (**C,D**) production in Luhan7 (LH-7) and Yangmai16 (YM-16) wheat cultivars. SS and MS treatments were applied at 35–40% and 55–60% soil field capacity (FC), respectively for ten days at tillering and jointing growth stages followed by re-watering, whereas WW was maintained at 75–80% FC. Time-course of the measurements was one day before stress (0DS), 5^th^ and 10^th^ day of stress (5DS, 10DS), 1 and 3 days after re-watering (1DRW, 3DRW). Shaded areas indicate the measurements taken following re-watering. Each vertical bar above the means indicates standard error of six replicates (*n* = 6) by using two-way ANOVA at P < 0.05.

The total soluble sugars (TSS) and fructose accumulation increased due to drought stress as compared to WW conditions (Fig. 8). The increase in TSS and fructose was higher under SS than MS treatments and this trend was more pronounced during tillering than during jointing. Sensitive plants showed less of an increase in TSS and fructose than did tolerant plants. After re-watering, the concentrations of TSS and fructose decreased and rate of recovery was lower under SS than MS treatments in both cultivars.

### Changes in leaf water relations and osmotic adjustment

Drought stress at both stages altered leaf water relations by decreasing leaf water potential (Ψw), osmotic potential (Ψs), turgor osmotic potential (Ψs^100^), and RWC but increasing osmotic adjustment (OA) as compared to WW plants (Table 2). During the drought periods, leaf water potential and magnitude of OA was lower in the sensitive cultivar than the tolerant cultivar. At the tillering stage, plants of both cultivars maintained higher water potential and relative water content and exhibited greater OA as compared to the jointing stage. After re-watering, stressed plants of both cultivars tended increase water potential and decrease OA values in both growth stages. Return of leaf water relations parameters to that of well-watered plants was more rapid in drought tolerant plants than sensitive plants, and differences between these cultivars were more pronounced at tillering stage than the jointing stage.

**Table 2:** Changes in leaf water relations and osmotic adjustment under drought stress and re-watering conditions during tillering and jointing growth stages of two wheat cultivars. The measurements were made at last day of drought stress and three days after re-watering.

Stress stages	Tillering						Jointing					
Water levels	SS		MS		WW		SS		MS		WW	
Parameters	LH-7	YM-16	LH-7	YM-16	LH-7	YM-16	LH-7	YM-16	LH-7	YM-16	LH-7	YM-16
Ψ_w_ (−MPa)	1.84b	1.95a	1.32d	1.54c	0.43e	0.41e	2.05b	2.25a	1.57d	1.65c	0.56e	0.51e
Ψs (−MPa)	2.56a	2.37b	1.87c	1.75d	1.25e	1.26e	2.45a	2.33b	2.05c	1.96c	1.43d	1.41d
RWC (%)	81.80d	77.50e	89.50b	86.40c	94.50a	94.80a	77.50c	73.80d	85.30b	83.40b	93.40a	94.00a
Ψs^100^ (−MPa)	2.09a	1.88b	1.65c	1.57d	1.18e	1.19e	1.89a	1.74b	1.71b	1.63c	1.33a	1.32a
OA (MPa)	0.91a	0.75b	0.51c	0.40d	—	—	0.56a	0.47b	0.41c	0.30d	—	—
Re-watering												
Ψ_w_ (−MPa)	0.81b	0.89a	0.53d	0.61c	0.43e	0.43e	1.10b	1.17a	0.74b	0.78b	0.57c	0.56c
Ψs (−MPa)	1.81a	1.76a	1.17b	1.10c	0.95d	0.95d	1.89a	1.83a	1.28b	1.21b	0.91c	0.90c
RWC (%)	91.10b	87.90c	93.50ab	93.00ab	94.50a	94.70a	86.50c	84.70c	92.30b	91.50b	93.10a	93.80a
Ψs^100^ (−MPa)	1.10ab	1.17a	1.03b	1.02b	0.93c	0.91c	1.63a	1.55b	1.18c	1.13c	0.84d	0.84d
OA (MPa)	0.65a	0.62a	0.19c	0.16c	—	—	0.73a	0.70a	0.33b	0.29b	—	—

Ψ_w_: leaf water potential, Ψs: osmotic potential, Ψs^100^: turgor potential, RWC: relative water content. Severe (SS) and moderate (MS) drought stress were applied at tillering and jointing, respectively, and followed by re-watering. WW indicated well-watered treatment. The shaded area indicates the measurements taken following re-watering. The lower case letters following the data represent significant differences for the means of six replications (*n* = *6*) under tillering and jointing stages, respectively, as measured by the Post hoc Test at *P* < *0.0*5 during each experiment season of 2014–2015 and 2015–2016.

### Effects of drought stress on crop phenology, dry matter and grain yield traits

The response of crop phenological, dry matter and grain yield traits to drought stress depended on the drought intensity, crop growth stage and the cultivar (Table 3). Moderate drought treatments applied both at tillering and jointing stages had little or no significant effect on days to anthesis (DA), grain filling duration (GFD), dry matter and grain yield reduction in both cultivars. However, severe drought stress treatments both at tillering and jointing significantly shortened the DA and GFD and reduced dry matter and grain yield traits including spike number per pot, grains per spike, 1000-grain weight and grain yield per pot. However, reductions in these growth and yield parameters under drought treatments were less in the tolerant cultivar as compared to the sensitive cultivar, and these reductions were more pronounced at jointing than tillering in both cultivars. Consequently, higher pre-drought limitation (PDL) and lower drought index (DI) values were recorded under SS than MS, and this trend was more pronounced in the sensitive cultivar than the tolerant cultivar. However, drought treatments increased harvest index in both cultivars when applied at both vegetative stages, indicating that drought during vegetative stages affected wheat performance mainly by reducing total dry matter accumulation.

**Table 3:** Changes in crop phenology, dry matter and grain yield traits under drought treatments when applied at tillering and jointing growth stages of two wheat cultivars.

Stress stages	Tillering				Jointing					
Water levels	SS		MS		SS		MS		WW	
Parameters	LH-7	YM-16	LH-7	YM-16	LH-7	YM-16	LH-7	YM-16	LH-7	YM-16
DA (days)	167.1d	164.2e	171.2bc	172.2b	165.2f	162.9g	170.2c	170.5c	173.1ab	173.7a
GFD (days)	37.4d	37.3d	39.5b	38.7c	36.3e	34.9f	38.1cd	37.5cd	40.1ab	40.9a
DM (g pot^−1^)	110.9d	102.6e	121.5b	118.8c	101.3e	87.5f	118.5c	109.5e	125.4ab	132.8a
PDL	12.0d	18.7c	3.2g	7.6e	21.2b	29.1a	6.6f	11.2d	—	—
Spikes (pot^−1^)	24.3d	26.6c	28.2c	31.6ab	26.6c	30.6b	26.6c	31.1b	27.3c	32.6a
Grains (spike^−1^)	35.0f	38.6d	34.8f	43.3b	31.8g	33.9f	35.0f	39.9c	36.4e	44.8a
1000-GWt (g)	45.8a	38.7e	45.1ab	39.3d	42.8c	34.6g	44.7b	36.7f	45.2ab	38.3e
Grain yield(g pot^−1^)	47.3b	43.1d	49.7a	49.7a	42.8d	38.9e	47.8b	45.6c	50.5a	51.1a
HI	0.42ab	0.42ab	0.40c	0.42ab	0.41b	0.43a	0.40c	0.41b	0.40c	0.38d
DI	0.93b	0.84d	0.98a	0.96a	0.84d	0.76e	0.94b	0.89c	—	—

DA, days to anthesis; GFD, grain filling duration; DM, total above-ground biomass production; PDL, pre-drought limitation; HI, harvest index, DI, drought index. Different lowercase letters following the data within the same row indicate significant differences among the means of five replications (*n* = *5*) at *P* < *0.05*. Severe (SS) and moderate (MS) drought stresses were applied at tillering and jointing, respectively, and followed by re-watering. WW indicates well-watered treatment.

## Discussion

Cultivar-specific metabolic responses were exhibited by wheat plants to cope with the effects of drought stress at the tillering and jointing stages of development. These differences in metabolic response to drought during vegetative development also resulted in altered crop phenology, dry matter accumulation, and final grain yield. Several drought tolerance mechanisms were employed by the crop and included ROS detoxification, maintenance of leaf water relations, and improved membrane stability were recorded, which enabled the wheat plants to evade lasting drought-induced damage, thereby allowing tolerant plants to more readily recover their physiological functions after re-watering. It was observed that plants upregulated ROS scavenging processes through enhanced antioxidant enzyme activity and increased content of non-enzymatic antioxidants and exhibited osmotic adjustment. However, the ability of wheat plants to maintain function during drought stress and recover after re-watering were dependent on the severity of drought stress, the crop growth stage at which drought occurred and the genotypic differences in drought tolerance.

Photosynthesis and stomatal conductance were decreased during the drought period, and these changes were reversed after re-watering (Fig. 2). It is clear from Fig. 2, that photosynthetic sensitivity of wheat plants to drought stress was mainly attributed to declines in stomatal conductance, which decreased CO_2_ availability to chloroplast and ultimately limited net photosynthesis. Similarly, after re-watering, the recovery of gs and Pn in tandem indicated that increases in stomatal aperture with duration of rewatering facilitated diffusion of CO_2_ from the atmosphere to the carboxylation site of Rubisco. Lower Pn and gs rates (Fig. 2) and less amount of water used by plant of drought-sensitive cultivar (Yangmai-16) (Table 1) indicate that this cultivar maintained less plant metabolic activities relative to Luhan-7 cultivar during the stress periods. Similarly, a lower decrease in Pn and gs as a consequence of drought stress and a higher degree of recovery observed at the tillering stage suggested that the tillering stage can more readily adapt to drought stress and recover when compared to jointing stage. When CO_2_ availability and carbon fixation are reduced, ROS form due to saturation of the electron transport system but limited availability end electron acceptors^6^. We observed an excessive accumulation of O_2_^•−^ and H_2_O_2_, leading to oxidative stress which caused an increase in MDA content, an indicator of oxidative damage to the membranes of stressed plants^18^. Higher ROS and MDA contents in the sensitive cultivar as compared to tolerant cultivar (Fig. 4) (P < 0.05) might be associated with greater photosynthetic inhibition under drought stress and an increased potential for ROS production. ROS capable of damaging the photosynthetic apparatus and cause oxidation of proteins, lipids, nucleic acids, and carbohydrates^7,8^. Our results showed that higher MDA concentration in drought-stressed plants was associated with higher H_2_O_2_ content and greater rate of O_2_^•−^ generation, especially in the severely drought-stressed plants.

Wheat plants displayed a suite of drought resistance and recovery traits to overcome the effects of oxidative stress. During the early days of drought, wheat plants showed a substantial increase in CAT and SOD activities. SOD converts O_2_^•−^ into H_2_O_2_ which is further catabolized by CAT to prevent oxidative damage^19^. APX also functions as a major enzymatic scavenger for H_2_O_2_^20^. During the stress period, the level of SOD and CAT increased immediately, but as the stress period prolonged to 10DS, their levels decreased (Fig. 5). This might be because these ROS scavengers are usually water soluble, and are destroyed during ROS detoxification or through self-oxidation. The cell has limited capability to re-synthesize the destroyed or oxidized scavengers during an extended period of stress^34^. Consequently, tissues become extremely prone to ROS attack under prolonged stress^21^. Cultivar-specific differences in antioxidant enzyme capacity may partially explain differences in tolerance as the sensitive cultivar exhibited less ability to increase and maintain antioxidant enzyme activity under drought stress, resulting in poorer recovery as compared to the more tolerant cultivar. As an example, the tolerant cultivar displayed enhanced antioxidant enzyme activity (SOD, CAT, and APX) and lower MDA accumulation Our results indicate that enhanced ROS detoxification promotes drought tolerance by decreasing oxidative damage to tissues, thereby facilitating greater recovery in more drought tolerant genotypes.

A lower increase in GSH concentration in the tolerant cultivar seems counterintuitive initially, but it is likely that upregulation of antioxidant enzymes is the dominant method of ROS detoxification in the tolerant cultivar, whereas the sensitive cultivar exploited GSH as an attempt to mitigate oxidative stress (Fig. 6A,B). A greater magnitude of increase in the concentration of GSH in sensitive wheat leaves relative to tolerant wheat leaves during drought is in accordance with the previous report on wheat given by Herbinger *et al*.^22^. GSH can serve as an antioxidant either by scavenging ROS directly like ascorbate or indirectly as a reducing agent to convert ascorbic acid from its oxidized form to its reduced form^10^. In addition to developing a greater pool of antioxidants, the tolerant cultivar maintained a higher level of photoprotective pigments (carotenoids) (Fig. 6C,D). Carotenoids perform an important role in heat dissipation of excess excitation energy in the photosynthetic apparatus, which helps prevent the initial formation of superoxide in plants receiving excess light energy as photosynthesis declines under drought conditions^6^. Thus, the carotenoids might have played an important role in restraining ROS accumulation in the chloroplasts via photoprotection of the photosystem.

After re-watering, the return of H_2_O_2_, O_2_^−^, and MDA concentrations in drought stressed plants to the level of WW plants indicated that wheat plants have the ability to tolerate and recover from water stress at the cellular level. Moreover, the activities of CAT, SOD, APX, and GSH remained slightly higher under severe stress but returned to level of WW plants under moderate stress after re-watering (Figs 5, 6), indicating that enzymatic and non-enzymatic antioxidants recovered to levels comparable to well-watered conditions, and that a steady-state level of ROS generation and scavenging rates was reached that minimized oxidative stress, which was also confirmed by lower MDA levels after re-watering (Fig. 4E,F).

Under drought stress, a higher accumulation of soluble sugars, free amino acids, and proline were recorded in this study (Fig. 7). It is suggested that these compatible solutes may aid in stress tolerance in wheat plants by improving osmotic adjustment, ROS detoxification, protein stabilization, and cell membrane protection^6^. As a result of the higher accumulation of osmolytes the osmotic potential of cells was decreased, which in turn facilitated diffusion of water into the cell, thereby maintaining a higher turgor potential (Table 2). The maintenance of favorable cellular turgor potential under water limited conditions allows the plant to maintain physiological functions such as stomatal opening, CO_2_ assimilation, and cell expansion and development^23^. Proline is considered the main component of osmotic adjustment and this osmolyte plays a key role in mitigating oxidative damage and stabilizing cell membranes^24^. These results are in agreement with the report of Yi *et al*. 2016^5^ who found that under drought stress, there was a progressive increase in free proline in cotton plants, and that of Monreal *et al*. 2007^25^ who reported a notable proline accumulation in sugar beet leaves when drought stress became severe and protein synthesis was diminished by giving rise to free amino acids. We observed that after re-watering, the proline and free amino acid concentrations returned to the values of WW plants. Under drought stress, the accumulation of proline and other amino acids and degradation of protein were inversely proportional to the water status of plants (Fig. 7), i.e., proline and amino acid production were correlated with a decrease in leaf water potential, suggesting the contribution of these solutes in osmotic adjustment.

In addition to being an integral component of osmotic adjustment, soluble sugars have been reported to be closely associated with the cellular antioxidation system^26^. Nishikawa *et al*. 2005^27^ also confirmed this relationship and found that high soluble carbohydrate production in the florets of broccoli enhanced ascorbate synthesis, which regulated ROS buildup in the chloroplast. Similarly, significant constructive effects of exogenously applied sugars have previously been reported in a herbicide-induced photo-oxidative stress remediation in Arabidopsis^28^. Data presented in Fig. 8 indicate that wheat plants responded to drought stress with increased sugar accumulation, which might have increased the ROS scavenging potential of wheat plants. So, it can be proposed that higher ROS levels in the drought sensitive cultivar of the current study might be associated with lower levels of soluble sugars since sugar starved plants have been reported to produce more ROS^29^. Additionally, after re-watering, a rapid reduction in sugar levels might be an indication of a quick breakdown of sugars upon relief from stress providing the plants with sufficient energy to repair damaged tissues. Therefore, it can be elucidated that the synergistic association of sugars with the cellular antioxidative system contributes to drought tolerance in wheat.

Phenological response to drought included earlier anthesis and maturity under severe drought stress (Table 3). Phenological adaptation is a well-developed drought-escape mechanism and a key determinant of grain yield in cereal crops^30^. The results showed that severe drought stress during vegetative stages particularly affected crop development, growth, and final productivity by altering plant physiological functions. The greater grain yield declines under drought stress in the drought-sensitive cultivar (Table 3), especially under severe stress might be associated with a greater decline in photosynthesis during stress and limited recovery after re-watering relative to the tolerant cultivar. The plants under severe stress exhibited earlier maturity with a shorter life cycle and had lower grain numbers and decreased weight per grain. The findings of lower final yields under drought stress during vegetative stages are in accordance with previous reports of Foulkes *et al*.^30^. Severe stress caused the plants to produce a small canopy and tended to senesce earlier, which hastened their life cycles and decreased grain number and weight.

## Conclusions

Wheat cultivars exhibited different metabolic features in terms of ROS accumulation, oxidative damage, antioxidant capacity, and production of osmotically active solutes under moderate and severe drought levels in the present study. In the current study there were two tolerance mechanisms employed in response to drought stress; the first involved the upregulation of antioxidant enzyme (SOD, CAT, and APX) activity and production of non-enzymatic (GSH and carotenoids) antioxidants; whereas the second involved accumulation of soluble sugars, free amino acids, and proline to facilitate osmotic adjustment. These metabolic allow the wheat plant to withstand and survive water-deficit conditions. The more drought tolerant cultivar exhibited greater photosynthetic stability during drought and more rapid recovery following drought, primarily due to greater ability to scavenge ROS and to osmotically adjust. Moreover, plants were better able to recover from stress imposed during the tillering stage than the jointing stage, emphasizing the importance of drought timing in determining productivity. These results revealed that the plant’s ability to maintain physiological functions during drought and recover after re-watering during vegetative periods are important for determining final productivity in wheat.

## Materials and Methods

### Plant culture and growth conditions

The experiment was carried out under a rain exclusion shelter during the growing seasons of 2014–2015 and 2015–2016 at Pailou Experimental Station of Nanjing Agricultural University (32^◦^04′N, 118^◦^76′E), China. The two most widely grown wheat cultivars in the region (lower Yangtze River Basin), namely Luhan7 (drought-tolerant) and Yuangmai16 (drought-sensitive) were used as experimental materials^2^. These cultivars show similar phenology and yield potentials under optimum field conditions. Uniform selected seeds of both cultivars were surface-sterilized by dipping in 0.5% hypochlorite solution for 20 min and then rinsing thoroughly with distilled water followed by drying before sowing. Fifteen surface sterilized seeds were planted in free-draining plastic pots of 1125 cm^3^ volume. Each pot was filled with 8 kg air-dried, sieved (2 mm) and uniformly mixed clay loam soil with 13% soil moisture. At the time of soil filling, 0.8 g N, 0.5 g P_2_O_5_ and 1.1 g K_2_O per pot were applied for each treatment. Further, 0.4 g per pot N splits were applied at jointing and booting, respectively. Thinning was carried out 10 days after germination to ten seedlings per pot. Then a week later, the second thinning was done and seven uniform seedlings per pot were retained for subsequent studies. Each pot was irrigated to 75–80% field capacity (FC) (leaf water potential was −0.50 to −0.70 MPa) with tap water having 7.5 pH, 1.2 dsm^−1^ electrical conductivity (EC) and 1200 mg L^−1^ total soluble salts (TSS) until the start of the stress treatments.

### Drought stress application and management

As shown under experimental design (Fig. 1), three soil water regimes consisting of a non-limiting soil water level (WW), moderate drought stress (MS) and severe drought stress (SS) corresponding to 75–80% FC (leaf water potential −0.50 to −0.70 MPa), 55–60% FC (leaf water potential of −1.20 to −1.40 MPa), and 35–40% FC (leaf water potential of −1.80 to −2.20 MPa), respectively were applied to each cultivar. Another factor included in the experimental design was timing of drought stress. Specifically, the stages tillering (40 days after planting, Feekes 2.0, beginning of tillering) and jointing (130 days after sowing equivalent to Feekes stage 6.0) were selected to apply the severe and moderate drought stress treatments on separate replicates. For drought stress imposition, the irrigation to pots was withheld until the soil FC reached to 55–60% and 35–40% for moderate and severe drought stress, respectively. Then drought stress treatments were maintained for 10 days. Soil FC for the specific drought level was maintained by weighing pots and then compensating the water lost by the addition of an equivalent amount of water and measuring the pre-dawn leaf water potential on a daily basis. During the stress period, control pots were maintained at 75–80% FC. Soil water status of the pots was measured before the water application, and the amount of water required for irrigation was calculated by the equation below:

1 $$W=D\times H\times A\times (FCI-FC0)$$

where, W is the amount of irrigation water, D is the soil bulk density, H is the soil depth, A is the area of each pot, FC1 is the desired soil field capacity, and FC0 is the actual soil field capacity before irrigation.

A factorial experimental arrangement of treatments was utilized according to a completely randomized design with two levels of cultivar (Luhan7 and Yuangmai16), three different water regimes (severe, moderate and well-watered) and two drought application stages (tillering and jointing) as independent variables of interest. Each treatment had 30 replicates for sampling and measurements.

### Plant sampling

The uppermost, fully expanded leaves from six to ten plants in each treatment were separated one day before starting drought stress (0DS), the 5^th^ and 10^th^ day of drought stress (5DS, 10DS), and 1 and 3 days after re-watering (1DRW, 3DRW) at both growth stages. Three sampled leaves were immediately put into liquid N for five minutes and then stored at −40 °C for the assays of ROS, enzymatic and nonenzymatic antioxidants. Six leaves for each treatment were used for the determination of leaf water potential (Ψ_w_), osmotic potential (Ψs), relative water contents (RWC), and membrane stability index. Other sampled leaves were oven dried at 70 °C for 72 h and powdered for the measurements of total soluble sugars, fructose and sucrose, proline and total free amino acid concentrations. Pots were only used once for sampling and measurements and then discarded from the experiment. Concomitant with destructive leaf samplings, leaf gas exchange measurements for net photosynthetic rate and stomatal conductance were also performed.

### Plant analysis and measurements

#### Measurements of leaf water relations and osmotic adjustment

Leaf relative water content (RWC) was determined according to the standard method proposed by Barrs and Weatherly, 1962^31^ as RWC = (FW − DW)/(TW − DW), where FW is fresh leaf weight, DW is dry weight and TW is turgid weight after 24 h floating in distilled water at 4 °C in darkness. Leaf water potential (Ψ_w_) was measured according to the method of Scholander, 1964^32^ using a pressure chamber (PMS Instrument Co., Corvallis, OR, USA). Osmotic potential (Ψs) was measured according to Sánchez *et al*.^33^. The leaves were put in a glass vial at −20 °C for 24 h. After thawing at room temperature, the cell sap was extracted with a gentle hand compress, and the Ψs was measured by using a vapor pressure osmometer (Wescor Vapor 5520, ELITech Group Inc., Logan, UT, USA). The turgid osmotic potential at 100% RWC (Ψs^100^) was determined by the equation: Ψs^100^ = Ψs × (RWC/100). Then osmotic adjustment (OA) was estimated as the difference in Ψs^100^ between well-watered plants (Ψs_ww_^100^) and the drought-stressed plants (Ψs_d_^100^)^34^

2 $${\rm{OA}}={\rm{\Psi }}{{\rm{s}}}_{{\rm{ww}}}^{100}-{\rm{\Psi }}{{\rm{s}}}_{{\rm{d}}}^{100}$$

### Membrane stability index and membrane injury

The membrane stability index (MSI) was measured by using a conductivity meter following the method of Khanna-Chopra and Selote, 2007^34^. Leaf samples of 200 mg were thoroughly washed in double distilled water and placed in two separate 10 mL tubes of distilled water. One tube was heated for 30 min at 40 °C in a water bath and electrical conductivity was measured (C1). The second set was boiled for 10 min at 100 °C in a boiling water bath and electrical conductivity was measured (C2). The MSI was estimated by the equation given below:

3 $${\rm{MSI}}=[1-(\frac{{\rm{C}}1}{{\rm{C}}2})]\times \,100\,$$

Membrane injury (MI) was estimated as ratio of MSI of drought-stressed plants and MSI of control plants as given by Dhanda *et al*.^35^;

4 $$\mathrm{MI}\,( \% )=[1-(\frac{{\rm{MSId}}}{{\rm{MSIc}}})]\times \,100\,$$

### Gas exchange measurements

The photosynthetic rates and stomatal conductance of individual leaf blades were measured between 9:00 and 11:00 a.m. using a portable photosynthesis system (Li-6400; LI-COR Inc., Lincoln, NE, USA). The youngest fully expanded leaf was placed in the leaf chamber at a photon flux density of 1000 µmol m^−2^ s^−1^; the flow rate through the chamber was 500 µmol s^−1^ and the leaf temperature was 25 °C. The ambient CO_2_ concentration was approximately 380 µmol CO_2_ mol^−1^ air, and the vapor pressure deficit was approximately 2.0 kPa. Each treatment included six replications.

### Determination of reactive oxygen species, lipid peroxidation, and enzymatic antioxidant activities

Reactive oxygen species, lipid peroxidation (estimated by measuring the malondialdehyde contents: MDA) and antioxidant activities in the leaf were determined following the methods given by Tang *et al*.^36^ and Zhang *et al*.^37^. Fresh leaf samples (0.5 g) were sliced and homogenized in a mortar and pestle with 5 mL ice-cold extraction buffer containing 50 mM potassium phosphate buffer (pH 7.0) and 0.4% polyvinylpoly pyrrolidone (PVP). The homogenates were centrifuged at 10,000 × g for 30 min at 4 °C. Then supernatants were collected and used as crude extracts for the above-cited assays by using a Pharmacia Ultra Spec Pro UV/VIS spectrophotometer (Pharmacia, Cambridge, England).

Superoxides (O_2_^•−^) were determined with a reaction mixture of 0.5 mL phosphate buffer (pH 7.8), 1 mL 1 mM hydroxylammonium chloride, 1.0 mL 17 mM P-aminobenzene sulfonic acid and 1.0 mL 7 mM α-naphthylamine. The mixture was incubated at 25 °C for 60 min and absorbance was noted at 530 nm. Hydrogen peroxide (H_2_O_2_) was determined after isolation by peroxidase coupled assay using 4-aminoantipyrine and phenol as donor substrates. The carbonyl content in oxidatively modified proteins was quantified using the 2,4-dinitrophenylhy-drazone assay procedure by recording the absorbance at 290 nm. Contents of MDA were determined through thiobarbituric acid (TBA) method at 532 nm, and then corrected by subtracting non-specific absorbance values at 600 nm by using an extinction coefficient of 156 mmol L^−1^ cm^−1^.

Superoxide dismutase (SOD) activity was determined according to Tang *et al*. 2010^36^ by adding 0.1 mL enzyme extract to a reaction mixture of 1.5 mL 50 mM sodium phosphate (pH 7.8), 0.3 mL130 µM methionine, 0.3 mL 750 µM nitro-blue tetrazolium (NBT), 0.3 mL 100 µM EDTA-Na_2_, 0.300 mL 20 µM riboflavin and 100 µL distilled water, and illuminated under light of 4000 flux for 20 min and then sample absorbance was determined at 560 nm. One unit of SOD activity was considered as the amount of enzyme used for 50% inhibition of the NBT reduction. Peroxidase (POD) activity was determined by adding 50 µL enzyme extract to a reaction mixture containing 1.0 mL 50 mM sodium phosphate (pH 5.5), 1.0 mL 0.3% H_2_O_2_ and 0.95 mL of 0.2% guaiacol. The absorbance value at 470 nm for changes in a unit of POD enzyme activity was noted. For catalase (CAT) activity, 200 µL enzyme extract was added to the reaction mixture of 1.5 mL 50 mM sodium phosphate (pH 7.8), 300 µL 0.1 M H_2_O_2_ and 1.0 mL distilled water. The change in absorbance at 240 nm per minute as a unit of CAT activity was recorded. For ascorbate peroxidase (APX) activity, 200 μL enzyme extract was added to a reaction mixture of 50 mmol L^−1^ potassium phosphate buffer (pH 7.0), 0.5 mmol L^−1^ ASC and 0.1 mmol L^−1^ H_2_O_2_. APX activity was determined by noting the decrease at 290 nm for 1 min in 1 mL of the reaction mixture.

### Determination of non-enzymatic antioxidants

For reduced glutathione (GSH) determination, 0.5 g wheat leaves were homogenized with 5 mL of 3% metaphosphoric acid and centrifuged the homogenate for 10 min at 10,000 × g^38^. For carotenoid content determination, 0.2 g frozen leaf samples were placed in a vial with 4 mL of dimethyl sulphoxide for 24 h in the dark for pigment extraction. Then samples were centrifuged at 5,000 × g for 15 min at 4 °C. The absorbance of the supernatant was measured at 664 nm to calculate the concentration of carotenoids^39^.

### Determination of carbohydrates, free amino acids, proline, and soluble protein concentrations

Total soluble sugars and fructose contents were determined spectrophotometrically from the soluble and residual fractions of ethanol-water extracts by following the methods described by DuBois *et al*.^40^. Total soluble sugar contents were determined at 620 nm using an anthrone reagent following by incubation at 90 °C. Fructose content was determined at 420 nm using concentrated H_2_SO_4_. Contents of total free amino acids and proline were determined at 570 and 520 nm, respectively by using a ninhydrin reagent as described by Bates *et al*.^41^. To determine the total soluble protein contents, 0.5 g frozen leaf samples were pestle and extracted by a buffer of sodium phosphate. The mixtures were centrifuged at 4,000 × g, 4 °C for 10 min. The supernatants were standardized using bovine serum albumin solution and then absorbance values were recorded spectrometrically at 595 nm.

### Determination of phenological changes, dry matter, and grain yield traits

The number of days from planting to anthesis was recorded when plants under a given treatment had reached 50% flowering. Similarly, grain filling duration was expressed as the number of days from anthesis to grain physiological maturity, defined as when 50% of spikes under a treatment had reached grain maturity. At maturity, whole plants from five randomly selected pots were severed at the soil surface using pruning-scissors and weighed to determine total above-ground biomass.

Pre-drought limitation (PDL) in above-ground dry matter production due to drought treatments was estimated according to Xu *et al*.^3^ as follows:

5 $$PDL( \% )=\frac{(DMC-DMT)}{DMC}\,\times \,100$$

where DMC is total dry matter in pots under WW conditions and DMT is the total dry matter under drought stress followed by re-watering.

Spikes in a pot were cut to count the number of spikes per pot, threshed individually by hand and final grain yield traits were recorded. Harvest index was calculated as grain yield fraction of total aboveground biomass.

6 $$HI( \% )=\,\frac{Grain\,yield}{Total\,above\,ground\,biomass}\times \,100$$

Drought index (DI) was estimated as grain yield differences between drought stress treatments as compared to control pots according to method of Huang and Zhao 2001^42^;

7 $$DI=\frac{YD}{YW}$$

where YD is the grain yield under drought stress treatments and YW is the grain yield under WW conditions.

### Data analysis

A two-way analysis of variance (ANOVA) was performed using the General Linear Model procedure to calculate the effects of drought stress and cultivar on metabolic parameters for each sampling and measurement point as well as for end of season growth parameters. Means were compared using Duncan’s multiple comparison tests for *post hoc* analysis (P ≤ 0.05) using the SPSS statistical package (SPSS Inc., Chicago, IL, USA). Figures were plotted by using Sigma Plot 10.0 software (Systat Software Inc., Chicago, IL, USA).
